# Chronic rhinosinusitis: a qualitative study of patient and clinician experiences of the MACRO randomised controlled trial of surgical versus medical management

**DOI:** 10.1136/bmjopen-2025-108999

**Published:** 2026-03-11

**Authors:** Jane Vennik, Clare McDermott, Samantha J Williams, Mike Thomas, Jim Boardman, Carl M Philpott, Paul Little, Anne Schilder, Claire Hopkins

**Affiliations:** 1 Primary Care and Populations Sciences, University of Southampton, Southampton, UK; 2 University College London Ear Institute, London, UK; 3 Norwich Medical School, University of East Anglia, Norwich, UK; 4 James Paget University Hospitals NHS Foundation Trust, Great Yarmouth, UK; 5 ENT, Guy's and St Thomas’ Hospitals NHS Trust, London, UK

**Keywords:** QUALITATIVE RESEARCH, Patient Preference, Endoscopic surgery, OTOLARYNGOLOGY

## Abstract

**Objectives:**

To explore patient and clinician experiences of participation in the MACRO randomised controlled trial (RCT)—which found endoscopic sinus surgery (ESS) to be clinically effective whereas clarithromycin was no better than placebo for chronic rhinosinusitis (CRS)—and to identify barriers and facilitators to the implementation of the trial results.

**Design:**

Qualitative study embedded within the multicentre MACRO RCT. Semistructured interviews with patients and clinicians were analysed using thematic analysis.

**Setting:**

21 secondary and tertiary ear, nose and throat centres in England and Scotland participating in the MACRO RCT.

**Participants:**

20 CRS patients (16 with nasal polyps, 4 without) were interviewed approximately 6 months after trial completion, and 17 clinical staff including principal investigators (PIs), associate PIs and research nurses.

**Results:**

This study explored patients’ and clinicians’ experiences of the trial to identify barriers and facilitators to implementing the findings. Adopting the outcomes of the trial would involve recommending surgery to more patients with CRS. Yet patient and clinician interviews highlighted polarised views on ESS among patients, between those with positive experiences and expectations of ESS and those expressing fear of complications and hesitancy to receive surgery. During the trial, many participants randomised to surgery reported rapid improvement in symptoms, but with postoperative challenges for some patients including pain, unexpected symptoms and variations in recovery period. Priorities for implementation include providing patients with information about risks and support to make informed choices. Clinicians also reflected on the resource implications for offering ESS to more patients.

**Conclusions:**

ESS is effective for CRS, but patient hesitancy and recovery concerns persist. Implementation requires clear communication, recognition and respect for individual preferences, tailored support for decision-making and post-surgical care to optimise acceptance and outcomes.

**Trial registration number:**

ISRCTN36962030.

**STRENGTHS AND LIMITATIONS OF THIS STUDY**

- This qualitative study was embedded within a large multicentre randomised controlled trial (MACRO), providing rich insights into patient and clinician experiences of trial participation and interventions.
- Purposeful sampling achieved diversity in patient characteristics (treatment allocation, nasal polyp status, age and site) and clinical roles, enhancing transferability of findings.
- Thematic analysis enabled exploration of patient experiences of the trial, and factors influencing future implementation.
- We did not include patients who declined MACRO trial participation, who may have held different views.
- Most participants were of white ethnicity, which may limit the transferability of findings to more diverse populations.

## Introduction

 Chronic rhinosinusitis (CRS), a prevalent inflammatory condition of the nasal and paranasal sinus mucosa, affects 9% of adults in the UK.^1^ Patients with CRS frequently present with symptoms of nasal obstruction, rhinorrhoea, facial pain/pressure and anosmia. Acute exacerbations and uncontrolled symptoms can have a significant impact on health-related quality of life which is comparable to that observed in other chronic conditions including congestive heart failure, angina and chronic obstructive pulmonary disease.^2^ The burden on NHS health services is significant, with 120 000 outpatient consultations for CRS and around 17 000 sinus surgeries conducted annually in England (National Consultation Information Programme (NCIP) portal data).

Primary care management of CRS typically involves intranasal medications and oral corticosteroids, prior to referral to ear, nose and throat (ENT) services where patients who fail appropriate medical treatments (including antibiotics) are prioritised for endoscopic sinus surgery (ESS).^3^ The MACRO trial was designed to address a need for clearer research evidence on the role of longer-term antibiotics and ESS in CRS management.^4^

MACRO was a randomised controlled trial which compared ESS, clarithromycin and placebo in CRS patients with and without nasal polyps.^5^ The trial was undertaken in 21 secondary and tertiary ENT centres in England and Scotland between 2018 and 2023. A total of 514 patients with CRS were randomised to ESS, clarithromycin or placebo and followed up for 6 months. The trial found that ESS was significantly better than clarithromycin or placebo at 6 months based on the patient-reported Sino-Nasal Outcome Test - 22 (SNOT-22)^6^ score, whereas clarithromycin showed no significant benefit compared with placebo.^7^

A qualitative process evaluation was conducted alongside the MACRO trial to explore clinician and patient experiences of recruitment, trial delivery, participation and trial interventions. The qualitative study aimed to understand barriers and facilitators to future implementation of trial findings by exploring the patient and clinician views of participation in the MACRO trial and experiences of undergoing ESS and medical management of CRS.

## Methods

### Design

This was a qualitative study nested within the MACRO randomised controlled trial,^5^ prospectively registered with the ISRCTN registry (36962030). The qualitative work was conducted prior to the analysis of the main MACRO trial.

The qualitative team comprised two post-doctoral researchers (JV and CM) and one postgraduate researcher (SJW), all female, together with a male general practitioner and researcher (MT). All were independent from the MACRO trial team being based at a different UK university. The qualitative team met regularly to discuss sampling, data collection and data analysis. This study is reported in line with Standards for Reporting Qualitative Research checklist^8^ (online supplemental appendix 1).

#### Participants and procedures

##### Recruitment of patient participants

MACRO trial participants who consented to be contacted were purposefully sampled to take part in a qualitative interview. Participants were sampled based on a range of characteristics including age, gender, trial recruiting site, nasal polyp status and treatment allocation (medical or surgical arm), and the selected participants were contacted by email. Once consent and eligibility were confirmed, a convenient date and time for interview was arranged.

##### Recruitment of clinician participants

Purposive sampling was used to ensure coverage across the MACRO recruiting sites, and to ensure inclusion of all clinical roles within the trial, male and female interviewees, and trial sites with high and low recruitment rates. Clinical roles included principal investigators (PIs) (consultant surgeons or consultant physicians responsible for leading the trial at their site), associate PIs (resident doctors enrolled on the National Institute for Health and Care Research (NIHR) Associate PI scheme) and research nurses (RNs). One interviewee was a clinical research practitioner, but to ensure anonymity when presenting quotes, they have been included within the RN category. Clinical staff were invited to take part by email with an attached participant information sheet. Interested participants responded directly to the qualitative team. All interviewees gave written informed consent for audio-recording and interview.

##### Interviews

The semistructured interview guides (patients and clinicians) were developed iteratively through discussions with clinicians and the trial management team, informed by previous qualitative work on the MACRO project^9 10^ (onlinesupplemental appendices 2  3).

Patient interviews were conducted after patients had completed the trial. Those randomised to the medical arm remained blinded to their treatment allocation (clarithromycin, placebo) as they entered long-term follow-up (for 5 years). Patients in the surgical arm were aware of their allocation. Clinician interviews were conducted towards the end of the MACRO trial and clinicians were similarly blinded to medical allocation but aware of surgical allocation.

Interviews were conducted over Microsoft Teams or by telephone to allow data collection with participants from different parts of England and were audio-recorded. RNs at two sites paired with a second RN colleague to co-participate in an interview. Interviews with patients were conducted by JV. Interviews with clinicians were conducted by CM, SJW and JV. Field notes were made during and following the interviews to note any observations relevant to interpreting the data (eg, emotional tone, non-verbal cues, background sounds, sound quality or any distractions). The interviews were transcribed verbatim by a university-approved professional transcriber in preparation for analysis.

###### Theoretical underpinnings and positionality statement

The philosophical approach to this study is one of subtle realism^11^ where we acknowledge that individuals (both clinicians and patients) experience things from their own perspectives, and as researchers we seek to interpret and understand these experiences.

The qualitative researchers were involved in the development of the MACRO trial and evaluation of the pilot phase but are neither clinicians nor patients. This positioned the researchers as outsiders to both the clinical and patient communities, in the role of independent inquirers rather than advocates. The researchers held no supervisory, evaluative or institutional power over the clinicians who participated in the study. This independence helped to reduce social desirability bias within interviews and minimise conflicts of interest.

### Analysis

Patient interviews were conducted and analysed before the staff interviews. Analysis was conducted in an iterative process, starting after the first few interviews were transcribed and continuing alongside data collection. We continued interviews until analysis indicated that the data was sufficiently rich and varied to answer the research question.^12^ The interview guide was refined as the interviews continued, guided by the evolving analysis. NVivo V.12 for Windows was used for data management and to facilitate analysis. A thematic analysis^13^ approach was used, drawing on methods of constant comparison. SJW, CM and JV independently coded the first interview and discussed and agreed preliminary codes. Discrepancies were minor, and codes were refined and adjusted to ensure that the final analysis achieved consistency. The agreed coding framework was then applied to the remaining transcripts and descriptive themes generated.

Clinician interviews were conducted and analysed separately following the same procedure, but early codes were informed by the coding framework and themes from the patient interviews. The final themes and subthemes from the patient and clinician interviews were then mapped together to provide a more detailed understanding and description of the MACRO trial. Combining clinician and patient data facilitates a more comprehensive and contextualised understanding of the trial, capturing perspectives from both those who delivered the MACRO trial and those who experienced it. It enables the identification of convergence and divergence views, to explore relational and contextual factors that might be missed if the groups were considered in isolation.

### Patient and public involvement

A patient and public involvement (PPI) panel comprising 10 CRS patients and members of the public, varied according to age, gender and clinical experiences of CRS, was involved in the design of the MACRO programme, application for funding, oversight of the trial and interpretation of trial findings. For this qualitative study, PPI members contributed to the overall study design and development, piloting of the interview topic guide and interpretation of the findings. In addition, one member of the PPI panel (JB) is a coauthor for this study. His contributions are detailed in the author’s section.

## Findings

### Participants

A total of 37 patients were contacted at approximately 6 months (1–10 months) after completing the MACRO trial, most of whom were participating in the 5-year trial follow-up period. Of these, 20 were purposefully sampled to take part in an interview between November 2022 and February 2023. Interviews ranged from 16 to 63 min (mean 30 min) in duration (table 1).

**Table 1:** Characteristics of patient participants

	Medical component	Surgical component	Total (n=20)
Phenotype			
Non-polyp	3	1	4
Polyp	6	10	16
Age			
31–40	2	4	6
41–50	1	1	2
51–60	4	2	6
61–70	2	3	5
71–80	0	1	1
Self-reported gender			
Male	7	7	14
Female	2	4	6
Demographic region of the UK			
North East	1	2	3
East	2	2	4
London	2	4	6
South East	3	2	5
Scotland	1	1	2
Ethnicity			
Asian or Asian British-Pakistani	1	0	1
White-British	6	6	12
White-Irish	1	0	1
White-other	0	1	1
No data	1	4	5

A total of 17 trial clinical staff agreed to take part in an interview between March and August 2023. Interviews lasted between 20 and 60 min (mean 36 min) (table 2).

**Table 2:** Characteristics of clinician participants

Healthcare professional characteristics	N=17
Self-reported gender	
Male	9
Female	8
Clinical role	
PI (consultant surgeon or physician responsible for trial at site)	4
Associate PI (resident doctor registered on NIHR Associate PI training scheme)	3
Research nurse	10
Demographic region of the UK	
North East	2
East	4
London	3
South East	4
South West	1
Scotland	3
Interview type	
Individual interview	13
Joint interview	4 (2 interviews with 2 research nurses in each interview)
Mean Interview duration	
Individual interviews	33 (20–60 min)
Joint interviews	37 (44–60 min)

NIHR, National Institute for Health and Care Research; PI, principal investigator.

### Descriptive themes

The analysis generated four descriptive themes relating to patient and clinician experiences of the MACRO trial, and one theme relating to patient and clinician views on implementing trial results.

1. Pathways to the MACRO trial.
2. Diverse treatment perspectives and the need for informed support.
3. ESS: rapid relief, postsurgical challenges.
4. Experiences of medical management: gradual improvement, manageable side effects.
5. Implementing trial results: barriers and facilitators.

Themes are presented and supported by verbatim quotes.

### Pathways to the MACRO trial 

#### Seeking a better future

To enter the trial, patients needed to be referred to an NHS ENT hospital department in the UK and to have already tried routine medications for CRS without success. Patients’ motivations to enter the trial included hope that participation might benefit their CRS, anticipation that taking part would involve enhanced care, and altruistic hopes that supporting research might help other patients in the future.

Trials, anything that I’m offered I’m happy to participate in, so long as I don’t feel like I’m going to lose a limb on it. Yes, somebody’ s got to do it, and I suppose from a personal point of view I have a sense that there will be a higher degree of care and ongoing assessment, I suppose. (P15, patient, medical allocation)

Clinicians reflected that the structured support and enhanced access to clinical care in the trial gave patients hope. Regular contact with healthcare professionals was particularly reassuring for participants.

One of the pros is they get to see the doctor regularly and have ENT input. *So, that’ s quite a draw.* (*Research Nurse, 12*)

Some patients had been attracted to the trial through the possibility that if randomised to surgery, they might receive this sooner than in routine care.

I was sat alongside of the computer, and I said, ’ Well, that’ s brilliant,’ because I know, in the outline documents, it says you’ll get that within six weeks. I said, ’ That’ s fantastic. I’ll go with that’ (P18, patient, surgical allocation)

While some patients hope that the trial might provide faster surgery, this was not a motivation for all, since some patients were hesitant about surgery or wanted to avoid it, as will be explored in theme 2.

#### Running out of options for a distressing condition

Many participants recounted their distress about the impact of CRS on their lives. This included disrupted sleep, loss of ability to taste and smell, facial pain/headaches and impaired breathing and congestion, often lasting for many years.

I was just in agony nearly every single day, headaches, facial pain and pressure (P1, patient, surgical allocation).

CRS symptoms often affected activities that were previously enjoyed and reduced patients’ quality of life. Many patients reported feeling low, frustrated or despairing about their symptoms.

It can sometimes make you feel down if you just have one of those days when you’re just sick of it (P16, patient, surgical allocation).

Clinicians reported that patients with long-standing and unresolved CRS symptoms often felt exhausted by conventional care pathways. Their willingness to enter the trial was shaped by a sense that they had nothing left to lose.

They were suffering and they wanted an option which was going to make them better (Principal Investigator, 07)*.*

### Diverse treatment perspectives and the need for informed support

#### Differing views on treatment

Differences between patients in their preferred treatment were a theme that recurred within patient and clinician interviews. Attitudes among patients to ESS appeared particularly polarised into ‘pro-surgery’ or ‘anti-surgery’, as this clinician observed.

I do quite a few studies where we’re comparing surgery against not having surgery, and we do get the common recurring things that people are either dead against surgery or they just want surgery because they see it as the ‘ be all and end all’. (Research Nurse, 05)

Reasons for patient hesitancy around surgery included perceptions of surgical risk, concerns about post-trial complications or recovery time, and difficulty taking time off work for an operation. In the following quote, a patient with nasal polyps explains their anxiety about risks from ESS.

It just felt, although [clinician] outlined a couple of the risks, one was, they might do some damage, the lining of the brain or something, I’m trying to remember rightly, He’d never done that, and he would never, if he felt that that was going to be an issue, he would not continue with the operation. So, to some extent I was reassured. There’ s always a nagging doubt that things will go wrong. (P19, patient, medical allocation)

Clinicians also recognised these concerns among patients which led to hesitancy in trial participation.

We’ve had a few people that are petrified of having surgery, either from bad experiences themselves or not having an operation before. One lady as well that said, “ If I had the operation I think my employer would be unhappy about me having to take time off”. (Research Nurse, 05)

Patients’ attitudes towards antibiotics tended to be less polarised than for surgery. Some patients expressed enthusiasm about the possibility of being randomised to the medical arm, although several patients perceived antibiotics to give short-term rather than long-term benefit. However, patients and clinicians both reported that where a patient had found antibiotics unsuccessful in the past, or had concerns about long-term antibiotics, this could lead to reluctance to participate in the trial.

They said that they are afraid, starting a new antibiotic treatment, just for their personal reasons, they’re not really keen in taking antibiotics long term. (Research Nurse, 03)

Clinicians reported that some patients found the idea of not being able to choose their treatment difficult.

There are, undoubtedly, patients who are not happy being randomised. They don’t like the phenomenon of randomisation and uncertainty (Principal Investigator, 07).

Clinicians described tailoring discussions with potential recruits to manage expectations and ensure that patients only joined if they were genuinely comfortable with being assigned to any of the treatment arms.

#### Balancing benefits and risks

Participants discussed weighing up the relative benefits and risks of surgery and antibiotics. When discussing risks, participants expressed concerns about the possibility that ESS could cause lasting damage if things went wrong, for example, to eyesight. Some patients had felt considerable anxiety about possible risks from surgery.

I think the risks played in my head too much. There was a lot of thinking about it, and I just couldn’t go through with it (P13, patient, surgical allocation).

A different kind of risk discussed was the possibility that surgery might not help symptoms, or if it did, that the effect would not last long.

The effects of it [previous surgery] lasted about a month and a half. So it was not successful at all, and that kind of influenced my thinking (P16, patient, surgical allocation)

However, in most cases, clear explanations by the clinical teams and perceived advancements in surgical techniques seemed to help alleviate these fears.

Potential benefits from surgery were highlighted by many patients, especially those with polyps, where the need for surgery to remove them in order to clear the nasal passages was well understood by patients.

We found that polyp patients were much more likely to want surgery, from my experience, just because of the visible nature of it. (Research Nurse, 02)

I couldn’t see how anything else, but surgery, would get rid of [the polyps] (P4, patient, surgical allocation).

Perceptions of risk from taking antibiotics were expressed by a few patients, specifically about the potential for antibiotic resistance, although the level of anxiety seemed low and did not appear to affect patients’ willingness to take part.

I think we need to be careful that we don’t overuse antibiotics and create that superbug resistance to antibiotics, which I guess is always a challenge (P10, patient, medical allocation)

Clinicians observed that patient decision-making for the trial involved thoughtful reflection on how the treatment options would impact their daily lives and often intersected with personal circumstances such as employment status and caregiving duties.

Quite a few of the patients are in their 30s and 40s, and a lot of those people say they can’t take time off work to do appointments (Research nurse, 05).

Clinical staff played a key role in helping patients to navigate the decision-making process by clarifying misunderstandings, providing reassurance and encouraging realistic expectations.

We just try and talk through any concerns of where they are undecided or why they have a certain preference, just see if we can answer any of those concerns. (Research Nurse, 05)

Participants generally described feeling that their questions and concerns had been answered by clinicians or by the Patient Information Sheet. They appeared well-informed about the study and demonstrated a good understanding of the aims and objectives of the trial, including comparison of different treatments.

There was a placebo option, the new tablet, the antibiotic I think it was they were trialling, and the surgery option. Just trying to find out what was the best way forward (P13, patient, surgical allocation).

Ultimately, this theme highlights the nuanced and highly personal nature of trial participation decisions, with good communication grounded in balancing hope, risk and practicality.

### ESS: rapid relief, postsurgical challenges

#### Trial logistics

At many sites, clinicians reported achieving the delivery of surgery within the specified 6-week time frame.

We were able to find a list to get them on. Generally, it wasn’t a problem scheduling them, to be honest (Associate Principal Investigator, 07)

However, at other sites, clinicians reported various systemic barriers including long waiting lists, competing priorities for theatre time and the challenge of prioritising research patients over routine cases who had been waiting longer.

So there was an internal conflict on my part for prioritising these patients over other patients who had been waiting longer for their surgery (Principal Investigator, 14)

#### Immediate benefit

Many of the patients in the surgical arm described rapid improvement in their CRS symptoms. Specifically, this included a reduction in pain, being able to breathe more easily and an improved sense of smell.

Just not have pain around here [pointing to forehead] was just absolutely brilliant (P1, patient, surgical allocation)

All of a sudden, I could start tasting and smelling again; all my Christmases came back at once (P8, patient, surgical allocation)

Several patients expressed how much their quality of life had been transformed by the surgery and spoke of the speed of improvement in their symptoms following ESS.

The operation definitely made an instant difference (P16, patient, surgical allocation).

#### Postsurgical symptoms

Some patients reported postsurgical symptoms, including pain, swelling and bleeding, but these were often considered to be an expected outcome of the surgical procedure.

I felt very congested, very sore and my nose did bleed quite a bit for a good few days, but I was told that was completely normal (P1, patient, surgical allocation).

Others experienced more significant issues such as infection, significant bleeding or delayed healing.

I developed some strong pain in my eyes, and I had an infection right after that (P11, patient, surgical allocation).

Several participants mentioned how their recovery did not match their initial expectations. Some anticipated quicker results or minimal discomfort but encountered a more prolonged recovery. However, others reported a smooth recovery with no major issues, especially with those who had prior surgeries and knew what to expect. They described feeling better relatively quickly after the procedure, with some noting that symptoms such as bleeding and discomfort were within the expected range.

I had no pain or discomfort or black eyes or anything that I was expecting, so it was really good (P16, patient, surgical allocation).

Aftercare and follow-up were described by patients and clinicians as an important part of the trial. Participants described the importance of pain management, nasal rinsing and follow-up visits to monitor progress. A few patients noted a lack of postoperative information, leading to confusion or stress about management of symptoms. Clinicians reported that postsurgical complications were minor and manageable and within normal post-operative care protocols. 

Overall, while experiences varied, common postoperative challenges included managing pain, unexpected symptoms and the length of the recovery period. Effective communication with healthcare providers and clear post-operative instructions seemed to alleviate some concerns.

### Medical management: gradual improvement, manageable side effects

#### Gradual improvement

Patients in the medical arm tended to report more gradual improvement in symptoms, with reductions in congestion and an improved sense of smell.

After a couple of weeks, I felt as though the symptoms had receded somewhat (P15, patient, medical allocation)

Improvements were also thought to be associated with use of daily sinus rinses, and it could be difficult to determine whether their improvement had been due to oral medications or nasal rinses, particularly since improvements tended to be relatively minor.

I felt that was more just to do with the continual rinsing of the sinuses that was helping the sense of smell rather than anything that was being done by the treatment that I was on through the trial (P10, patient, Medical allocation).

Other patients reported limited or no substantial improvement in symptoms or quality of life. Some reported persistent nasal congestion and loss of smell and expressed disappointment with modest or lack of improvement. The lack of improvement and absence of side effects led some to speculate that they were in the placebo arm.

I would have put money on me being on the placebo route, because I literally felt no difference at all (P10, patient, medical allocation).

Experiences of trial medication could also be masked by other co-existing conditions.

I’d had quite a severe asthma attack, literally a few days before. I sounded like the trial wasn’t working, which was a bit frustrating, because I thought I’d felt really good (P3, patient, medical allocation)

#### Compliance with trial medications

Patients understood the importance of good compliance with their trial medication and good compliance was reported by most patients in the medication arm.

I think you have to take things seriously, really, because, otherwise, what’ s the point? (P3, patient, medical allocation).

Patients reported that the instructions for usage were clear. Many used processes to remind them to take their medication, including making part of their daily routine, using electronic reminders and to-do lists, and keeping medication in a visible place.

I just take it as a daily routine (P12, patient, medical allocation).

A small number of patients reported missing an occasional dose, but overall compliance appeared to be good within the trial. Clinicians agreed that patients adhered well to the medication regimen, and some appeared to show improvements, particularly those with nasal polyps.

#### Side effects

Gastrointestinal symptoms were the most commonly reported side effect by patients in the medical arm. For some, this could be an indication that they were taking clarithromycin, while others reported no side effects at all.

It felt very strong, but it did feel like it was actually doing something (P3, patient, medical allocation).

It had no side effects, and there was no improvement (P19, patient, medical allocation).

Clinicians concurred with patients that clarithromycin was generally well-tolerated, with few side effects that were mostly gastrointestinal.

Yes, there were a few [side effects]. It was GI side effects, in the main (Principal Investigator, 14)

### Implementation of findings: facilitators and barriers

Within the interviews, we explored clinicians’ views on the implementation of the MACRO trial results. Since interviews were conducted before trial results were revealed, we asked them to reflect on the potential outcomes (superiority of ESS; superiority of clarithromycin; no difference) and likely effect on CRS management. Subsequently, the MACRO trial results found ESS to be significantly more effective than clarithromycin or placebo. We therefore focus on this outcome.

#### Facilitators to implementation

Most clinicians reported that ESS was already used routinely as a mainstay of CRS treatment for those patients where medications had not worked, so routine practice would not significantly change with the trial reporting ESS being superior to medical treatment. However, the implications of ESS as a more effective treatment could mean that clinicians could justify offering surgical intervention for CRS to more patients, or to patients earlier in their illness trajectory.

So I don’t know how much of a difference it will make across the ENT/rhinology community, but, yes, if surgery is shown to be much—again, it depends on the degree, but if it is much more significantly effective, then I don’t really see—apart from those patients that aren’t eligible for surgery because of comorbidities, etc.—why you’d even think about prescribing [clarithromycin]. Just surgery as an early treatment would be the sensible choice, I think. (Principal Investigator, 19)

Effectiveness of surgery would likely lead to changes in the conversations with patients about surgery since there would be clearer evidence on benefits.

PI: I think it will clarify matters for us as clinicians. It will remove some of the uncertainty around consent to patients and whether the antibiotic is really useful or not. (Principal Investigator, 07)

ESS as a more effective treatment within the MACRO trial was not considered to have any major personnel, cultural or training shifts within ENT departments since relevant staff were already trained in and familiar with this intervention.

Considering patient perspectives, our interview data indicated that ESS was a recognised and generally understood treatment for CRS. Some of our interviewed patients had received ESS in the past while others had not, but most appeared familiar with the rationale and general procedures involved. For some interviewed patients, ESS was an acceptable, indeed preferred treatment for CRS. For others, concerns about safety and post-surgical complications remained a reason for hesitancy. The acceptability of ESS as a treatment therefore varied considerably between patients.

#### Barriers to implementation

Clinical resources, including operating theatre space and trained surgical staff, were seen by clinicians as the main barrier to implementing MACRO results. Among clinicians, opinions on the impact on waiting lists differed, with some suggesting little effect, since ESS was already their preferred intervention, while others expressed concern about adding to already lengthy surgical waiting lists.

Waiting lists are going to go up. We know that’ s not in a good state, anyway. So, I suppose if it’ s found to be a better option, the bonus is, it’ s a day surgery, but waitlist would go up, ultimately. (Research Nurse, 06)

Patient data suggested that patient concerns around surgical risks or post-operative pain/complications may affect uptake of ESS. While the MACRO results provide justification for offering ESS to more patients where this is clinically indicated, our findings suggest that there is likely to be a significant subgroup of patients who remain hesitant about surgery.

Throughout the interviews, the key importance of understanding and balancing risks and benefits when making decisions about treatments was underlined by patients and clinicians. Listening to and addressing patient concerns and providing clear and easily understandable information remains a priority emphasised by patients and clinicians alike, so that patients can make informed treatment decisions that fit their needs.

### Applying the RE-AIM framework to our findings

We used the RE-AIM framework^14^ to reflect on and organise our findings and to assist in identifying key implications. The RE-AIM framework recommends evaluating five interactive components (reach, effectiveness, adoption, implementation and maintenance) to better understand the factors that promote or inhibit the implementation of an intervention.

In terms of effectiveness, clinicians viewed the superiority of ESS as strengthening the existing evidence base, reducing uncertainty and enabling clearer discussions with patients about best management. Adoption was facilitated by the fact that ESS was already embedded within routine CRS management in the UK ENT profession, with clinicians, departments and surgical teams possessing the necessary skills, infrastructure and cultural familiarity to deliver the intervention, meaning that no major training or workforce changes were anticipated. However, future implementation would be constrained by resource-related barriers, particularly limited operating theatre capacity and existing surgical waiting lists, which some clinicians felt could be exacerbated if ESS were offered earlier or to a broader patient group without additional NHS investment. In consideration of the reach of the intervention, ESS was widely recognised and acceptable to some patients; however, concerns about surgical risk, postoperative pain and recovery acted as important barriers for others, potentially limiting uptake among a substantial subgroup despite clinical eligibility. Finally, maintenance of intervention delivery would be supported by the alignment of trial findings with current practice, suggesting sustainability over time. However, this would be contingent on ongoing capacity within surgical services and continued attention to patient-centred communication to balance risks and benefits and support informed, preference-sensitive decision-making.

## Discussion

### Summary

This embedded qualitative study identified important insights into the decision-making process for MACRO trial participation and the CRS patient pathway. On inclusion in the trial, patients had already tried routine medications for CRS without success, so many were frustrated with current available treatments and pathways and were keen to try other options, reflecting the experiences of many CRS patients. There was heterogeneity in preferences for treatment, but good clinical discussions and the possibility of receiving faster or alternative treatment options within the trial motivated participation. Patients in the surgical arm reported rapid improvement in symptoms, but common postoperative challenges included pain management, unexpected symptoms and a lengthy recovery period. Patients receiving medical management reported more gradual or limited symptom improvement, relying more on intranasal medications to manage their symptoms. Trial findings (ESS as clinically effective treatment for CRS) could be implemented within UK ENT without significant changes to workforce, training, infrastructure or culture, but may be limited by surgical capacity, existing waiting lists and patient concerns about surgical risks. This highlights the need for adequate resources and patient-centred communication.

### Comparison with the literature

Our study found that patients with severe, fluctuating and uncontrolled symptoms seek a rapid solution to their CRS. Current management pathways take a stepwise approach, typically starting with self-management using over-the-counter treatments, progressing to primary care management and referral for those with inadequate symptom control. EPOS guidelines^3^ recommend ESS for CRS with and without nasal polyps, but until now, evidence has been limited. ESS can be perceived by patients as offering a quick treatment option with a mechanism of action that is clearly understood, especially by those with nasal polyps, and the next step in their management pathway. Similar findings were reported in a qualitative study of CRS of patient perspectives of disease control, who found that patients are more willing to escalate treatment from self-management and medical management to surgery during acute exacerbations and uncontrolled disease.^15^

Despite this, our study found that patients can be hesitant or opposed to having surgery, especially if they have had poor prior experiences of the procedure itself, or if symptom relief had been short-lived. This is consistent with our previous qualitative work which identified hesitancy for surgery as a potential limitation to recruitment to the trial.^9 16^ Patients can be concerned about the surgery itself, including risks and postoperative complications.^17^ Others can have doubts over the effectiveness of ESS, poor prior experiences or disruption to daily routine/time off work. Clinicians need to recognise that there is heterogeneity in patient views, and that patients and clinicians may view things in different ways. This aligns with the work by Saydy *et al*^18^ who identified the importance of exploring patient goals and expectations as these do not always align with the clinician’s view. This has implications for communication between healthcare providers and CRS patients when deciding on best management. Patients need evidence-based information and support to help with decision-making about treatment and compliance with the treatment plan. The impressive improvements seen in the surgical arm of the study in patients undergoing ESS by appropriately skilled surgeons will allow improved information provision and enable effective communication in future clinical practice.

Patient expectations of surgery are variable and multifactorial: some patients in the study describe ESS as a temporary solution that they expect to require further treatment and potentially revision surgery in the future. Others, however, speak of surgery as ‘curative’ and a permanent fix to their CRS. Patients can be dissatisfied with surgery when it doesn’t meet their outcome expectations.^18^ Clinicians should work with patients to encourage realistic expectations of ESS. This will improve their postoperative recovery experiences and satisfaction with care.

As the MACRO trial results feed into national and international CRS management pathways, consideration needs to be given to the heterogeneity of patient views, taking into consideration their perceptions of symptom severity and control, concerns and hesitancy for surgery, and outcome expectations. Clinicians must meet patient information needs to ensure they are supported with their decision-making, help them comply with post-operative instructions which will lead to better outcomes and satisfaction with care.

### Strengths and limitations

This study provided insights into patient experiences of participating in the MACRO trial and of the trial interventions. Using purposeful sampling, we included a diverse range of participants ensuring that we captured the views and experiences from the three trial interventions from a wide range of recruiting ENT sites. However, we did not include CRS patients who declined participation in MACRO who may have held different views and had an underrepresentation of people from a non-white background which reflected the MACRO trial population. Additionally, the median time from when the patients completed the MACRO trial to taking part in an interview was 6 months. This was to ensure that the interviews did not affect the patient-reported primary outcome of the trial. However, it is likely that some participants may have forgotten some of their trial experiences, but we hope that the most relevant experiences were recalled.

### Conclusions

The MACRO trial results demonstrate that ESS is effective for CRS, but patient hesitancy and recovery concerns persist. Implementation may involve an increased role for ESS in the treatment of CRS but requires clear communication, recognition and respect for individual preferences, tailored support for decision-making and postsurgical care to optimise acceptance and outcomes. Implementation is also likely to require additional NHS resources in the short term to avoid lengthening waiting lists for surgery.

## Supplementary material

10.1136/bmjopen-2025-108999 online supplemental file 1

10.1136/bmjopen-2025-108999 online supplemental file 2

10.1136/bmjopen-2025-108999 online supplemental file 3

## Data Availability

Data are available on reasonable request.
