# Supplementary material for: Chronic rhinosinusitis: a qualitative study of patient and clinician experiences of the MACRO randomised controlled trial of surgical versus medical management
Source: BMJ Open. 2026 Mar 11;16(3):e108999. doi: 10.1136/bmjopen-2025-108999 (PMC12983716; doi:10.1136/bmjopen-2025-108999)
Supplement: online supplemental file 2 [file bmjopen-16-3-s002.docx]

**Appendix 2**

**Interview topic guide for MACRO trial patients**

**Can you tell me about your chronic rhinosinusitis (CRS) and how it affects you?**

**Can you tell me how you found out about the MACRO Trial?

What did you understand to be the purpose of the trial?**

- What did you think about the information that you were given about the MACRO Trial?

**Why did you decide to take part in the MACRO trial?**

**What did you think about the information that you were given about the trial?**

**Could you describe how it was decided what treatment you would get?**

**How did you feel about the treatment that you were allocated to?**

**For those who are allocated to the medical treatment arm:**

**You were allocated to the group who received medication. What were your thoughts about that?**

- Did you know or could tell which group you were in?
- What are your views about antibiotics for CRS?

**Did you experience any side effects with taking the medication?**

**Did you take your medication as recommended?**

**How effective do you think the medication was for your CRS?**

**For those who are allocated to the surgical treatment arm:**

**You were allocated to the group who received surgery. What were your thoughts about that?**

- Can you tell me a bit about how the surgery went?
- How quickly did it take place?

**Can you tell me about how you felt after surgery and your recovery?**

**How effective do you think surgery was for you?**

- How do you think surgery works for CRS?
- What are your views about surgery now that you have had it?

**You were provided with some intranasal medications for your CRS during the trial. Can you tell me what you thought of using them and any problems you had?**

**You were asked to attend a further 2 assessment visits at the hospital – after 3 and 6 months. Could you tell me how these visits went?**

**You were asked to complete online questionnaires after 6 weeks, 3 and 6 months. How did this work for you?**

**Could you tell me what has happened with your treatment now the trial has finished?**

**What are your overall thoughts of taking part in the trial?**
