# Supplementary material for: Chronic rhinosinusitis: a qualitative study of patient and clinician experiences of the MACRO randomised controlled trial of surgical versus medical management
Source: BMJ Open. 2026 Mar 11;16(3):e108999. doi: 10.1136/bmjopen-2025-108999 (PMC12983716; doi:10.1136/bmjopen-2025-108999)
Supplement: online supplemental file 3 [file bmjopen-16-3-s003.docx]

**Appendix 3**

**Interview topic guide for Clinicians**

**Could you tell me how you came to be involved with MACRO and what your role is within the trial?**

- - What do you think about the training that you received for the MACRO trial?
  - Support/information from the research team?

**Could you tell me about the patient referral pathway for CRS in your trust?**

- - What kind of diagnostic procedures would that normally involve?

**Please talk me through how you would usually treat patients with CRS**

- - Any challenges
  - Have these views changed during the course of the trial?
  - Polyps vs non polyps?

**When did recruitment start at your site and how has it been so far?**

- - What do you see were the main challenges to recruitment?
  - How has COVID affected things at your site?

**We are interested in understanding a bit more about patients who decided not to take part in MACRO. Could you tell me some of the reasons why some declined?**

**Could you tell me about the trial processes at your site**

- Who conducted the assessments, sniffin sticks etc.

**For those who are allocated to the medical treatment arm:**

- - How did patients react to being randomised to the medical treatment arm?
  - How were the trial medications dispensed/provided at your site.
  - Did patients report any side effects with taking the medication?
  - As far as you are aware, did participants take medication as directed?

**For those who are allocated to the surgical treatment arm:**

- - How did patients react to being randomised to surgery?
  - Can you tell me about how surgery went for those participants allocated to that group?
  - Did patients report any problems with surgery or recovery afterwards?
  - Timing of surgery in routine care compared with trial

**Patients were provided with intranasal medications during the trial. How effective do you think they were for CRS?**

- - Was the use of intranasal medications similar in both treatment groups?

**How does the delivery of the interventions compare with routine clinical care at your hospital?**

**What are your overall thoughts about being involved in the MACRO trial?**
