# Supplementary material for: Chronic rhinosinusitis: a qualitative study of patient and clinician experiences of the MACRO randomised controlled trial of surgical versus medical management
Source: BMJ Open. 2026 Mar 11;16(3):e108999. doi: 10.1136/bmjopen-2025-108999 (PMC12983716; doi:10.1136/bmjopen-2025-108999)
Supplement: Supplementary file 4 [file bmjopen-2025-108999.reviewer_comments.pdf]

PEER REVIEW HISTORY

BMJ Open publishes all reviews undertaken for accepted manuscripts. Reviewers are asked to complete a checklist review form and are provided with free text boxes to elaborate on their assessment. These free text comments are reproduced below.

ARTICLE DETAILS

Title (Provisional)

Chronic Rhinosinusitis: A Qualitative Study of Patient and Clinician Experiences of the MACRO Randomised Controlled Trial of Surgical vs Medical Management.

Authors

Vennik, Jane; McDermott, Clare; Williams, Samantha J; Thomas, Mike; Boardman, Jim; Philpott, Carl M; Little, Paul; Schilder, Anne; Hopkins, Claire

---

VERSION 1 - REVIEW

**Reviewer**	**1**
**Name**	**Ryu , Gwanghui**
**Affiliation**	**Sungkyunkwan University School of Medicine**
**Date**	**11-Oct-2025**
**COI**	**None**

This is a well-intentioned qualitative process evaluation embedded within the landmark MACRO RCT, directly examines patients' decision-making to participate in RCTs.

The paper remains methodologically sound but analytically shallow. Although 16 patient responses and 10 clinician responses are cited in the text, it is difficult to regard the overall dataset as being well-represented in the manuscript. Even considering that the data were derived from oral, interview-based narratives, the presented quotations and analysis do not sufficiently reflect the full range of interview material.

Compared with the previous study (reference no. 8), much of the content appears to overlap, except for the section discussing decision-making regarding participation in the clinical trial.

Its academic writing style is uneven, and large sections read like an internal report rather than a peer-reviewed scholarly article. eg. On inclusion in the trial, patients had already received (failed) maximal medical therapy, so many were frustrated with current management.

Minor comments:

The references require updating, particularly for those marked as "accepted for publication" (e.g., Lancet 2025 – accepted for publication).

Please provide the full term and a more detailed description of the composition and role of the PPI panel.

The role of JB, who appears to have been part of the PPI panel, should be clarified. It would be more appropriate to describe JB's contribution in the Author Contributions section and remove the detailed mention from the main text to maintain consistency with standard authorship conventions.

**Reviewer**	**2**
**Name**	**Zurynski, Yvonne**
**Affiliation**	**Macquarie University, Australian Institute of Health Innnovation**
**Date**	**15-Oct-2025**
**COI**	**None**

This was an interesting paper, I feel the reporting of results in the abstract could be much more rigorous and the theoretical underpinnings and methodological approaches appear to be inconsistently applied. The results need to be more clearly linked with the objectives - it's not at all clear what barriers and facilitators to post trial implementation were identified. A realist approach is not evident in the results nor in the conclusions. You have simply reported qualitative outcomes. Findings from the interviews of clinical staff seem to be omitted.

Given that this study is an adjunct to a clinical trial, more details about the trial are needed to provide context - how many sites were there, UK only or international? How many patients were recruited?

Page 6 of 24 line 27 consider gender rather than male and female

Provide additional information about the interviewers - who were they and what training did they have? Who interviewed the clinicians? What was the positionality and power-structure between interviewer and interviewee?

Please explain what was recorded in the field notes and why, and how was this information used in the analysis. Was it just what interviewees said or something else? e.g. their demeanor, reluctance to answer etc?

(page 6 of 24 line 53) Please clarify what you mean by "reflections" is - a preliminary analysis of key themes? Reflexivity in qualitative thematic analysis has a very different meaning to what has been implied in this paper.

If you are really using reflexive thematic analysis I would expect to see a positionality statement for all analysts and an exploration of how that positionality may have influenced interpretation.

In table 2 - PI is not a sufficient description - PIs can be clinician researchers or other types of researchers please provide additional detail is a little vague.

Why are you reporting sites for the patients but you report demographic regions for the clinicians (Tables 1 and 2)? Please harmonise.

Also in table 2 is 4 joint interviews are mentioned - is this one joint interview with 4 participants? or 4 joint interviews?

The statement ".. the data was considered sufficiently rich" (p6 of 24) How was this decided? Was there an iterative analysis of interviews as data was collected?

Greater justification is needed for combining the clinician interviews and patient interviews together at the outset. Was there any attempt made to see whether clinicians' interviews resulted in different themes to those of patients?

In fact, the analysis appears to be very strongly focused on patients only including in the first sentence describing the 5 themes (p9 of 24). The clinician's voices are minimal throughout the analysis - I would suggest more clearly bringing out clinicians' views or clearly state that this paper is focused on patients - which appears to be the current focus.

Although the paper promises to tell us about barriers and enablers of implementation there is remarkably little discussed about implementation barriers, enablers or strategies. No implementation outcomes are mentioned at all - e.g. acceptability, appropriateness feasibility, cost etc. You do talk about patient compliance with medications and at home procedures but that is not "implementation".

An attempt has been made to use realist theory to construct CMO configurations, but the CMOs are currently presented separately for medical, surgical and intranasal medication. A greater synthesis across these areas would provide a broader understanding of the complexities that patients and clinicians must deal with when implementing the outcomes of the trial. The "bit-by-bit" analysis doesn't come together at the end - consider working up a model as the contexts, mechanisms and outcomes you describe are not completely separate- they overlap and intersect. Perhaps consult with a senior realist evaluation researcher to help with this.

Grammar, referencing, etc.

The accepted paper in the Lancet needs to be properly referenced and must appear in the reference list as (accepted for publication). There are several acronyms that have not been defined e.g. PPI, RNTNE and some minor grammatical issues. More recent references are needed to justify the theoretical underpinnings and the analytical approaches the authors claim to have applied.

VERSION 1 - AUTHOR RESPONSE

Reviewer: 1	Response to reviewers
Dr. Gwanghui Ryu , Sungkyunkwan University School of Medicine	
**Comments to the Author:**	
This is a well-intentioned qualitative process evaluation embedded within the landmark MACRO RCT, directly examines patients' decision-making to participate in RCTs.<br><br>The paper remains methodologically sound but analytically shallow. Although 16 patient responses and 10 clinician responses are cited in the text, it is difficult to regard the overall dataset as being well-represented in the manuscript. Even considering that the data were derived from oral, interview-based narratives, the presented quotations and analysis do not sufficiently reflect the full range of interview material.<br>Compared with the previous study (reference no. 8), much of the content appears to overlap, except for the section discussing decision-making regarding participation in the clinical trial.	We acknowledge the need to minimise overlap with our previous paper, and to focus more findings specific to the current study. To address this, the rewrite<br>- Includes a new theme (Theme 5) focusing on barriers and facilitators to implementation.<br>- Condenses the sections which overlap most with our previous paper, whilst retaining some to give context and a clear story<br>- expands the section on decision making with additional quotes from clinicians<br>- Provide more detailed quotes from participants to illustrate findings.
Its academic writing style is uneven, and large sections read like an internal report rather than a peer-reviewed scholarly article. eg. On inclusion in the trial, patients had already received (failed) maximal medical therapy, so many were frustrated with current management.	We have redrafted the text to make the writing style more consistent, both between different sections of the paper.
**Minor comments:**	
The references require updating, particularly for those marked as "accepted for publication" (e.g., Lancet 2025 – accepted for publication).	Reference to published paper added.
Please provide the full term and a more detailed description of the composition and role of the PPI panel.	More detail provided in PPI section.
The role of JB, who appears to have been part of the PPI panel, should be clarified. It would be more appropriate to describe JB's	This has been updated.

contribution in the Author Contributions section and remove the detailed mention from the main text to maintain consistency with standard authorship conventions.	
Reviewer: 2	
Dr. Yvonne Zurynski, Macquarie University	
**Comments to the Author:**	
This was an interesting paper, I feel the reporting of results in the abstract could be much more rigorous and the theoretical underpinnings and methodological approaches appear to be inconsistently applied. The results need to be more clearly linked with the objectives - it's not at all clear what barriers and facilitators to post trial implementation were identified. A realist approach is not evident in the results nor in the conclusions. You have simply reported qualitative outcomes. Findings from the interviews of clinical staff seem to be omitted.	Revised version provides<br>- Abstract has been reworded with more detail and greater attention to rigor.<br>- Results section provides greater integration of patient and clinician findings and more quotes from clinicians.<br>- We have added a new theme focusing specifically on barriers and facilitators to implementation.<br>- We present our findings as a thematic analysis and have removed the abductive reasoning.<br>- We have added further details from the clinician interviews.<br>- We have removed theme 5 (Intranasal medications) as we consider this less relevant to the implementation of the trial findings, and overlap with previous research.
Given that this study is an adjunct to a clinical trial, more details about the trial are needed to provide context - how many sites were there, UK only or international? How many patients were recruited?	Details of trial included as requested in the introduction and the published paper now referenced.
Page 6 of 24 line 27 consider gender rather than male and female	Amended
Provide additional information about the interviewers - who were they and what training did they have? Who interviewed the clinicians? What was the positionality and power-structure between interviewer and interviewee?	Amended
Explain what was recorded in the field notes and why, and how was this information used in the analysis. Was it just what interviewees	We have added more details to this section for clarification.

said or something else? e.g. their demeanour, reluctance to answer etc?	
(page 6 of 24 line 53) Please clarify what you mean by "reflections" is - a preliminary analysis of key themes? Reflexivity in qualitative thematic analysis has a very different meaning to what has been implied in this paper.	This section has been revised for more clarity.
If you are really using reflexive thematic analysis I would expect to see a positionality statement for all analysts and an exploration of how that positionality may have influenced interpretation.	We have added a positionality statement to the Methods section
In table 2 - PI is not a sufficient description - PIs can be clinician researchers or other types of researchers please provide additional detail is a little vague.	We have added clarification of this term as requested.
Why are you reporting sites for the patients but you report demographic regions for the clinicians (Tables 1 and 2)? Please harmonise.	Amended - both tables now report demographic regions rather than sites.
Also in table 2 is 4 joint interviews are mentioned - is this one joint interview with 4 participants? or 4 joint interviews?	We have clarified this as requested in the table and also added more explanatory detail to the text in the Interviews section.
The statement ".. the data was considered sufficiently rich" (p6 of 24) How was this decided? Was there an iterative analysis of interviews as data was collected?	We have added more detail to clarify this as requested.
Greater justification is needed for combining the clinician interviews and patient interviews together at the outset. Was there any attempt made to see whether clinicians' interviews resulted in different themes to those of patients?	Justification added line 207 - 211
In fact, the analysis appears to be very strongly focused on patients only including in the first sentence describing the 5 themes (p9 of 24). The clinician's voices are minimal throughout the analysis - I would suggest more clearly bringing out clinicians' views or clearly state that this paper is focused on patients - which appears to be the current focus.	We accept the reviewer's comments and our redraft has aimed to bring out clinicians' views more clearly in the paper, including additional quotes from clinicians and a section reflecting on similarities and differences among patient and clinician views.

Although the paper promises to tell us about barriers and enablers of implementation there is remarkably little discussed about implementation barriers, enablers or strategies. No implementation outcomes are mentioned at all - e.g. acceptability, appropriateness feasibility, cost etc. You do talk about patient compliance with medications and at home procedures but that is not "implementation".	We have added a new Theme 5. focussing specifically on barriers and facilitators to implementation.<br><br>Reflection in discussion section on how findings relate to REAIM framework
An attempt has been made to use realist theory to construct CMO configurations, but the CMOs are currently presented separately for medical, surgical and intranasal medication. A greater synthesis across these areas would provide a broader understanding of the complexities that patients and clinicians must deal with when implementing the outcomes of the trial. The "bit-by-bit" analysis doesn't come together at the end - consider working up a model as the contexts, mechanisms and outcomes you describe are not completely separate- they overlap and intersect. Perhaps consult with a senior realist evaluation researcher to help with this.	We accept the feedback that the CMO configurations either need to be further developed to achieve greater synthesis or to be taken out.<br>After consideration we have removed this aspect of the paper and focussed on implementation
Grammar, referencing, etc.	
The accepted paper in the Lancet needs to be properly referenced and must appear in the reference list as (accepted for publication). There are several acronyms that have not been defined e.g. PPI, RNTNE and some minor grammatical issues. More recent references are needed to justify the theoretical underpinnings and the analytical approaches the authors claim to have applied.	Amended as requested.

VERSION 2 - REVIEW

Reviewer 1

Name Ryu , Gwanghui

Affiliation Sungkyunkwan University School of Medicine

Date **02-Feb-2026**

COI

I appreciate the authors' dedication to enhancing the manuscript. I find the changes and explanations generally adequate.
